# Evaluation of the Association between Sjögren Syndrome and Thyroid Cancer Risk: A Systematic Review and Meta-analysis: Sjögren Syndrome and Thyroid Cancer Risk

**DOI:** 10.31661/gmj.v14i.3753

**Published:** 2025-10-22

**Authors:** Mansour Salesi, Shadi Botshekan

**Affiliations:** ^1^ Isfahan University of Medical Science, Isfahan, Iran

**Keywords:** Sjogren’s Syndrome, Sicca Syndrome, Thyroid Neoplasms, Thyroid Carcinoma, Thyroid Adenoma, Thyroid Cancer

## Abstract

**Background:**

Sjögren’s syndrome affects the skin, joints, lungs, kidneys, liver, and thyroid. This research was aimed to assess the association of Sjögren’s syndrome with the thyroid carcinoma risk.

**Materials and Methods:**

The present study was used a systematic review and meta-analysis method. This study searched the databases ProQuest, PubMed, Web of Science, Cochrane, and the search engine Google Scholar until July 7, 2024. The level of significance was considered as P0.05, and all data analyses were done in STATA 14 software.

**Results:**

A review of 11 studies revealed that Sjögren’s syndrome increased the thyroid carcinoma risk in all patients (OR: 2.08, (95%CI: 1.47, 2.94)), in patients aged 40 to 49 years (OR: 1.43, (95%CI: 1.23, 1.67)), 50 to 59 years (OR: 4.65, (95%CI: 1.87, 11.58)), 60 to 69 years (OR: 1.34, (95%CI: 1.08, 1.66)) and in women ((OR:1.83, (95%CI: 1.35, 2.48). However, there was no significant association between Sjögren’s syndrome and thyroid carcinoma risk in men ((OR: 1.49, (95%CI: 0.95, 2.34). Moreover, patients with Sjögren’s syndrome who had a follow-up period of = 5 years ((OR: 1.68, (95%CI: 1.10, 2.54) and patients with a follow-up period of 5 years ((OR: 5.77, (95%CI: 1.97, 16.97) were at risk of thyroid cancer. Moreover, the thyroid carcinoma risk was in Europe ((OR: 3.26, (95%CI: 1.24, 8.56) and in Asia ((OR: 1.87, (95%CI: 1.27, 2.74). Primary Sjögren’s syndrome also significantly increased the thyroid carcinoma risk ((OR: 2.37, (95%CI: 1.44, 3.90).

**Conclusion:**

Sjögren’s syndrome increased the thyroid carcinoma risk, and female gender, fifth decade of life, European race, and involvement duration of more than 5 years were the exacerbating factors.

## Introduction

Primary Sjögren’s syndrome (pSS) is a common systemic autoimmune disease characterized by chronic inflammation and dysfunction of the salivary and lacrimal glands [1][2]. In this disease, the patient faces many personal and social issues. Therefore, it can affect the mental health of the patient and the social health of the society. For this reason, despite the high costs incurred for the diagnosis and treatment of the disease, it can cause a decrease in the quality of life and depression in patients [3]. In pSS, extra glandular organ systems such as lungs, kidneys, small vessels, and other endocrine glands are also implicated [4]. Sjögren’s syndrome is associated with many diseases, including an increased risk of non-Hodgkin’s lymphoma[5]. But regarding extra glandular manifestations, pulmonary complications rank at the top as having a higher prevalence in pSS compared to secondary type [6][7], ranging from 9-75% according to the diagnostic method used, including small airway disease, parenchymal alteration, and interstitial involvement [8].

Among the pulmonary manifestations of pSS, interstitial lung disease (ILD) is by far the most common [9][10][11], with prevalences ranging between 8% and 39.1%. ILD causes airway obstruction in patients [12][13]. Various factors contribute to the development of ILD [14]. Differences in prevalence can be due to various factors, including diagnostic criteria, environmental factors, race, the presence of underlying disease, and other factors [15][16][17]. The most frequently encountered subtype of interstitial lung disease is non-specific interstitial pneumonia (NSIP), whereas other sorts are usual interstitial pneumonia (UIP), lymphocytic interstitial pneumonia (LIP), and organizing pneumonia (OP) [18][19][20][21]. Interstitial lung disease is a common extraglandular complication related to survival in patients with pSS, which lowers the quality of life of those affected and serves as one of the frequent causes of premature death [22][23][24][25]. Conversely, systemic autoimmune diseases have been postulated to increase the risk of interstitial lung disease as reported in previous studies [26]. The prevalence of ILD in pSS patients has been reported differently and has been studied in different populations with heterogeneous factors. Therefore, systematic reviews and meta-analyses can provide more accurate and reliable evidence on this issue. Given that limited studies have been conducted in this area, and systematic reviews can evaluate all factors affecting the frequency of ILD, we investigated this issue in this study.

## Materials and Methods

### Study Protocol

This study was designed to evaluate the prevalence of ILD in patients with pSS. The PRISMA protocol [27], which is specific for systematic review and meta-analysis studies, was used and registered on the PROSPERO website (CRD42024566698).

### PICO Components

Population: Studies investigating the frequency or prevalence of ILD in patients with pSS were evaluated. Intervention: Not applicable. Comparison: Not applicable. Outcomes: The primary outcome was the prevalence of ILD in patients with pSS. Secondary outcomes included the prevalence of NSIP, UIP, LIP, and OP in patients with pSS.

### Search Strategy

To access resources, international databases ProQuest, PubMed, Web of Science, Cochrane, Embase, and the search engine Google Scholar were searched until July 1, 2024, without any time and place restrictions, using keywords (Primary Sjogren’s Syndrome, Primary Sicca Syndrome, Prevalence, "Lung Diseases, Interstitial", Interstitial Lung Disease, Interstitial Pneumonia) and their MeSH equivalents. Keywords were combined with operators (AND, OR), and an advanced search was performed. The list of resources of selected studies in the electronic search phase was manually reviewed. The search strategy in the PubMed database is mentioned in this section: ((Primary Sjogren's Syndrome OR Primary Sicca Syndrome) AND ("Lung Diseases, Interstitial" OR Interstitial Lung Disease OR Interstitial Pneumonia)) AND (Prevalence)

### Inclusion and Exclusion Criteria

Studies that had investigated the prevalence of ILD in patients with pSS were included in the meta-analysis. The following studies were excluded from the meta-analysis: studies where the type of Sjögren’s disease was not specified - letters to the editor - case reports - duplicate studies - studies where their data were incomplete - review studies - studies that did not have full text - studies with poor quality.

### Qualitative Assessment

Two authors evaluated the studies with the Newcastle Ottawa Scale checklist. In this checklist, a star system was used, such that a maximum of one star was given for each question, and only the question related to comparison had the possibility of allocating two stars. Therefore, the lowest score on the checklist was zero (lowest quality), and the highest score was ten (highest quality). The cut-off point in this study was considered six [28].

### Data Extraction

Two authors independently performed data extraction, and disagreements were resolved by a third researcher. From each study, information such as author’s name, type of study, sample size, country, number of women and men, year, average age, prevalence of ILD in patients with pSS in all individuals, prevalence of ILD in women with pSS, prevalence of ILD in men with pSS, prevalence of ILD subgroups in patients with pSS was extracted.

### Statistical Analysis

Metaprop implements procedures which are specific to binomial data and allows computation of exact binomial and score test-based confidence intervals. It provides appropriate methods for dealing with proportions close to or at the margins where the normal approximation procedures often break down, by use of the binomial distribution to model the within-study variability or by allowing Freeman-Tukey double arcsine transformation to stabilize the variances. Sensitivity analysis was also used to determine the most influential studies resulting from the current meta-analysis. Subgroup analysis and meta-regression were used to investigate the sources of heterogeneity, and the Funnel plot was used to assess publication bias. The I2 index has three classifications (<25% indicates low heterogeneity, between 25% and 75% shows moderate heterogeneity and >75% indicates high heterogeneity). Due to the moderate heterogeneity, this study applied a random-effects model. Data analysis was performed with STATA 14 software (developed by Computing Resource Center in California) , and the significance level of the tests was considered P<0.05.

## Results

**Table:** Table1. Characteristics of Articles

**References**	**Index**	**Continent**	**Place**	**Type of Sjogren's Syndrome**	**Type of study**	**Total number**	**Mean age**	**Duration of study**	**Follow-up (Year)**	**Risk of thyroid cancer**	**Low limit **	**Up limit **
**Park SK, 2024 ** **[20] **	HR	Asia	South Korea	NR	Cohort	17752	60-69	2002 to 2019	9.49	1.48	0.91	2.39
**Yang TH, 2023** **[22] **	OR	Asia	Taiwan	NR	Cohort	194650	40-49	between January 2012, and December 2019	NR	1.43	1.23	1.67
**Jia Y, 2023 ** **[21] **	OR	Europe	Europe	NR	Mendelian randomization	1080	NR	NR	NR	0.88	0.7	1.1
**Zhou Z, 2022** **[23] **	SIR	Asia	China	NR	Cohort	1329	50-59	between January 2006 and April 2015	6.65	8.41	4.34	14.68
**Isık OO, 2022 ** **[26] **	SIR	Europe	Turkey	Primary	Cohort	151	50-59	between 2004 and 2019	10.5	23.07	4.8	67.4
**Goulabchand R, 2021** **[27] **	HR	Europe	France	Primary	Cohort	278204	60-69	from 2011 to 2018	3.96	1.73	1.07	2.79
**Kang J, 2020 ** **[28] **	SIR	Asia	South Korea	Primary	Cohort	5482661	60-69	between January 2012 and December 2014	NR	1.19	0.87	1.52
**Ahn JK, 2020** **[29] **	SIR	Asia	South Korea	Primary	Cohort	NR	50-59	between 2007 and 2017	3.1	1.23	0.88	1.68
**Brito-Zeron P, 2017 ** **[19] **	SIR	Europe	Spain	Primary	Cohort	1300	50-59	2005-2016	7.58	5.05	1.89	13.45
**Weng MY, 2012** **[18] **	SIR	Asia	Taiwan	Primary	Cohort	7852	50-59	from 2000 to 2008	3.5	2.56	1.4	4.3
**Theander E, 2006 ** **[30] **	SIR	Europe	Sweden	Primary	Cohort	194	50-59	from 1984 until 31 December 2002	8	6.86	0.17	38.21

**Figure-1:**
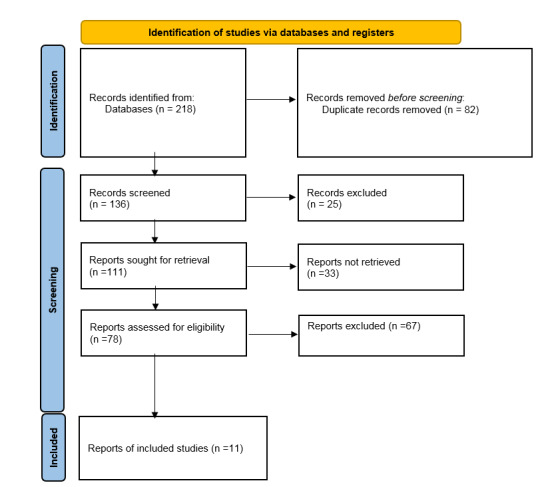
Figure 1. Chart of PRISMA

**Figure-2:**
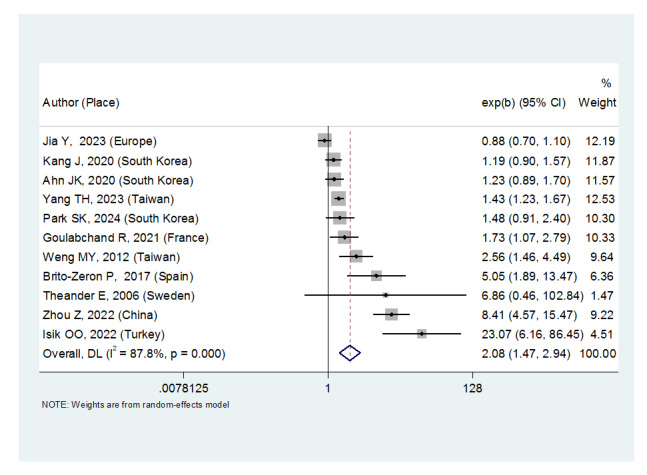
Figure 2. Forest plot related to the association between Sjogren's syndrome and thyroid cancer risk

**Figure-3:**
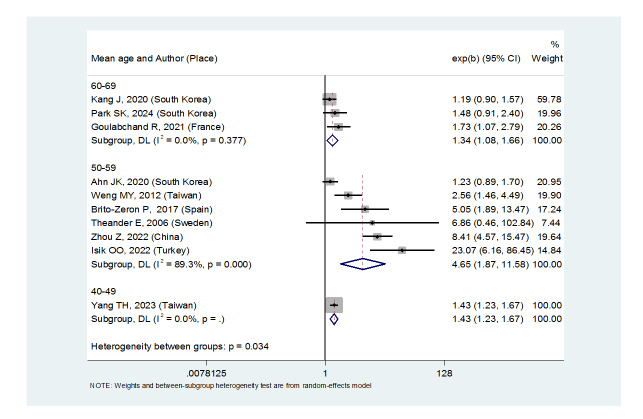
Figure 3. Forest plot related to the association between Sjogren’s syndrome and thyroid cancer risk by mean age

**Figure-4:**
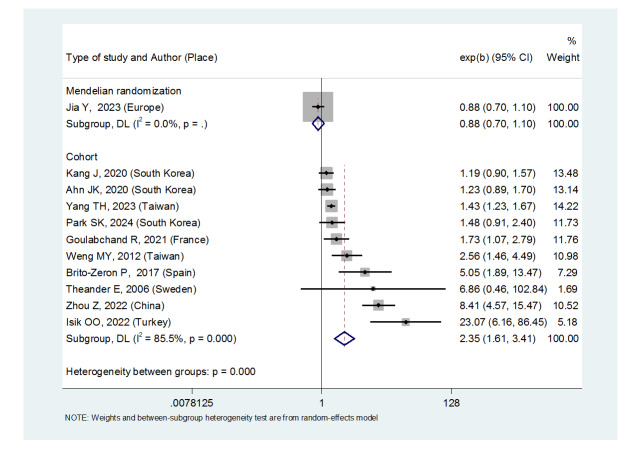
Figure 4. Forest plot related to the association between Sjogren's syndrome and thyroid cancer risk by type of study

**Figure-5:**
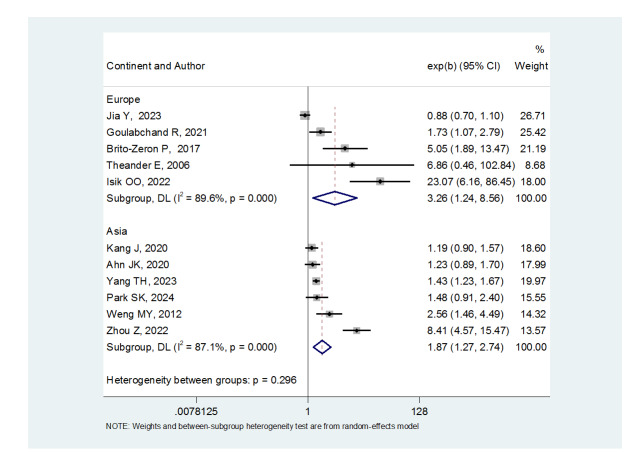
Figure 5. Forest plot related to the association between Sjogren's syndrome and thyroid cancer risk by continent

**Figure-6:**
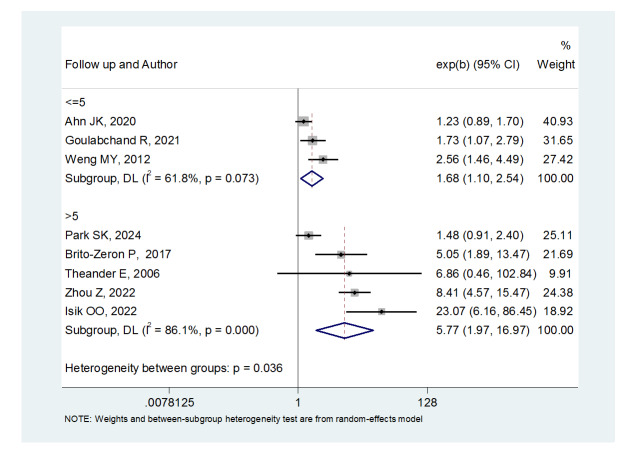
Figure 6. Forest plot related to the association between Sjogren's syndrome and thyroid cancer risk by follow up

**Figure-7:**
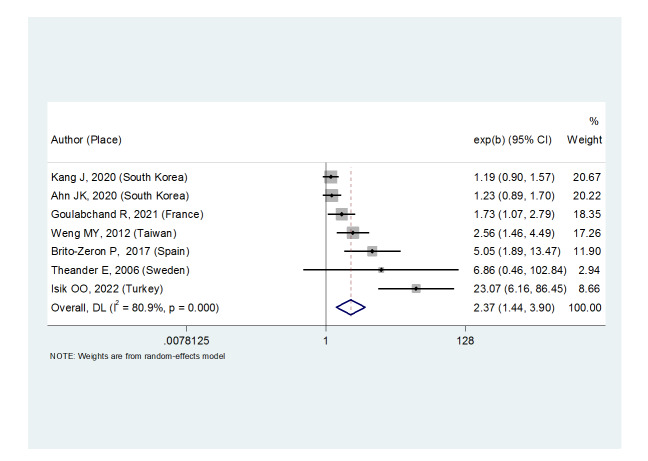
Figure 7. Forest plot related to the association between primary Sjogren's syndrome and thyroid cancer risk

Study Selection

There was a total of 302 studies at the initial stage. The study titles were screened, and 142 duplicate studies were removed. Abstracts of studies were assessed, and we excluded 14 studies because they did not have full text. The full text of the remaining 61 articles was evaluated; then41 studies without essential data were excluded. Seven further studies were excluded based on other items from the exclusion criteria, and 34 articles entered the systematic review and meta-analysis (Figure-1). This meta-analysis included 34 observational studies involving a population sample of 9535 individuals. Of these 34 studies, 26 were cohort studies, 5 were case-control studies, and 3 were cross-sectional studies. The studies were published from 1985 to 2024 (Table-1).

When excluding the studies of Sahin Ozdemirel [40] and Kvarnstrom [54], which were considered outliers, the overall prevalence estimate for ILD in pSS patients was 23.5% (95%CI: 18.6%, 28.4%) (Figure-2A). Furthermore, the prevalence of ILD in women with pSS was 31.2% (95%CI: 18.4%, 44.1%), and in men was 45.5% (95%CI: 23.6%, 67.4%) (Figures-2B and -2C). Consequently, the incidence of ILD in male patients with pSS was higher than in female patients, and the male gender could be considered an independent risk factor for developing ILD. As shown in Figure-2D, the subgroup analysis according to age detected a prevalence of ILD in patients with pSS:

• Age 30-39 years: 22.2% (95%CI: 18.9%, 26%)

• Age 40-49 years: 10.8% (95%CI: 8.7%, 12.9%)

• Age 50-59 years: 27.6% (95%CI: 17.9%, 37.4%)

• Age 60-69 years: 23.3% (95%CI: 17.9%, 28.7%)

Age was not statistically significantly associated with pSS-associated ILD, and the highest prevalence of ILD was in the age group of 50 to 59 years.

Regarding study design, the prevalence of ILD among pSS patients in:

• Case-control studies was 15.6% (95%CI: 7.1%, 24.1%)

• Cohort studies was 25.7% (95%CI: 19%, 32.4%)

• Cross-sectional studies was 21.4% (95%CI: 11.9%, 31%) (Figure-2E)

In patients with pSS, the prevalence of NSIP was 48.8% (95%CI: 41.4%, 56.2%), the prevalence of UIP was 15% (95%CI: 10.5%, 19.5%), the prevalence of LIP was 10.8% (95%CI: 6.6%, 15%), and the prevalence of OP was 9.4% (95%CI: 5.4%, 13.4%). Therefore, the most common type of ILD in patients with pSS, in order, was NSIP, UIP, OP, and LIP (Figures-3A-D). Figure-4 shows a meta-regression, indicating there was no statistically significant relationship between the prevalence of ILD in patients with pSS and the sample size of the studies (P=0.608). Moreover, Figure-5 demonstrates that the relationship between the prevalence of ILD in patients with pSS and the year of publication of the studies was not statistically significant (P=0.841). The publication bias diagram was significant (P=<0.001) and showed that studies that reported a high prevalence of ILD in patients with primary Sjögren's syndrome had a lower chance of publication (Figure-6). Sensitivity analysis showed that the studies by Wang Y (2018) and Kvarnstrom M (2015) were the most influential studies in the current research results (Figure-7).

**Figure-8:**
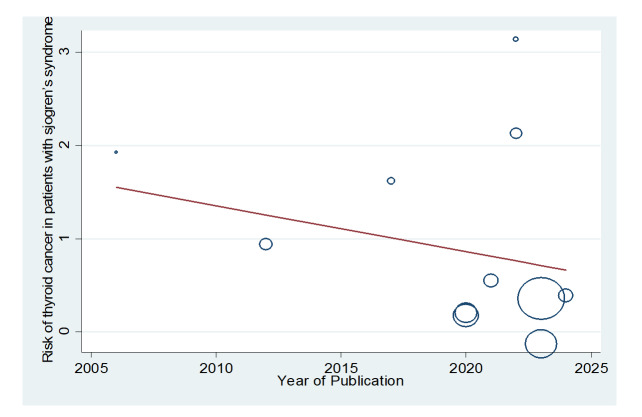
Figure 8. Meta-regression plot of the relationship between “association between Sjogren's syndrome and thyroid cancer risk” year of publication

**Figure-9:**
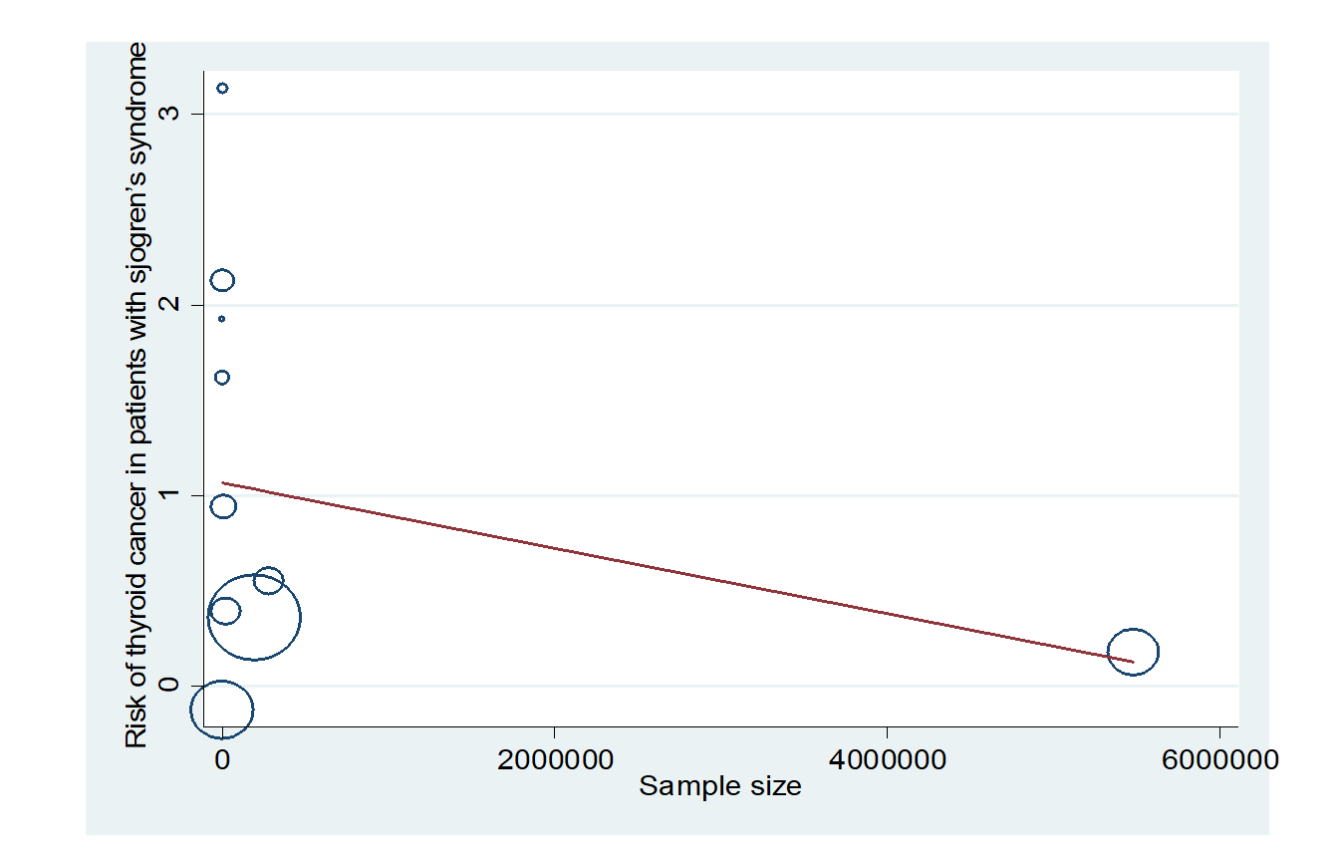
Figure 9. Meta-regression plot of the relationship between “association between Sjogren's syndrome and thyroid cancer risk” and sample size

**Figure-10:**
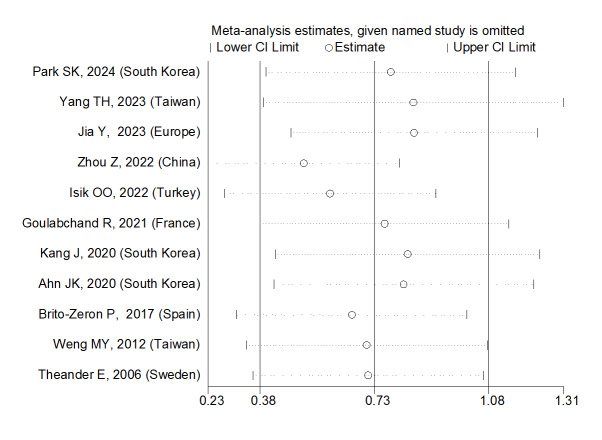
Figure 10. Sensitive analysis

**Figure-11:**
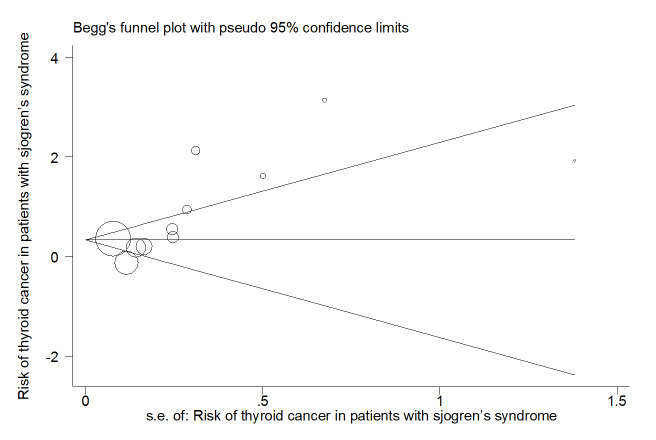
Figure 11. Publication bias diagram

## Discussion

In this meta-analysis, 34 observational studies were combined, and a total of 9535 patients with pSS were examined. The results revealed that the prevalence of ILD in all patients with pSS was 23.5%, and in women and men, it was 31.2% and 45.5%, respectively. Moreover, the prevalence of ILD in patients aged 30 to 39 years was 22.2%, 40 to 49 years was 10.8%, 50 to 59 years was 27.6%, and 60 to 69 years was 23.3%. In terms of types of ILD, the prevalence of NSIP in patients was 48.8%, UIP was 15%, LIP was 10.8%, and OP was 9.4%. In the current meta-analysis, NSIP was the most common type of ILD. In another study, the authors estimated a combined prevalence of 52% for NSIP and 44% for UIP in patients with pSS [60], which is consistent with the results of our study.

In 2024, in a cohort study conducted by Manikuppam and colleagues, which included 550 patients with pSS with an average age of 50 years, the prevalence of ILD was estimated to be 6%. This study was retrospective and the average age of the patients was close to 50 years. On the other hand, all patients were evaluated from one center. Therefore, these factors could be influential in the discrepancy between the results and the results of the present study [29].

In a study conducted by Lin and colleagues in 2022 on 333 patients with pSS, a retrospective study showed that the prevalence of ILD was 19.82%. In this study, it was found that the prevalence of ILD was higher in Asian populations than in Europeans. In contrast, in the present study, Iranian races were evaluated [35]. In another cohort study conducted by Guo and colleagues in 2021 on a large Chinese group consisting of 563 patients with pSS and 172 patients with secondary Sjögren’s syndrome, the prevalence of ILD in patients with pSS was 42.6% [38]. In a case-control study conducted by Gao and colleagues in 2018 on 853 patients with pSS, the aim of which was to investigate the prevalence, risk factors, and prognosis of ILD in patients with pSS, ILD was observed in 165 patients (19.34%) [19]. In another study conducted by Huang and colleagues in 2023 using a case-control method, the analysis of 274 patients with pSS showed that the prevalence of ILD was 22.3% [32]. Furthermore, due to the wide range of prevalence of ILD in patients with pSS, a systematic review and meta-analysis was required to perform. In some studies, the prevalence of ILD in patients with pSS was low, but on the other hand, in some studies, we faced a high prevalence of ILD in patients with pSS.

The difference in the prevalence of ILD in different studies compared to the present study could be influenced by various factors. These factors include geographical regions, race, age of patients, type of study, diagnostic criteria, and other factors. Therefore, factors can influence the frequency of ILD in different studies.

In a meta-analysis conducted in 2022 by Arbiv and colleagues to investigating the prevalence and radiological patterns of ILD in Sjögren’s syndrome, the prevalence of ILD was about 15% [61]. In the current meta-analysis, in line with the previous study, the prevalence of ILD in patients with pSS was 25. Although in the current study, only patients with pSS were examined, while in the previous study, patients with primary and secondary Sjögren’s were examined together.

In a systematic review conducted in 2020 by Sambataro and colleagues, the results showed that about 20% of pSS patients have ILD [ 62]. In a meta-analysis conducted by He and colleagues in 2020, in 23 studies with 6157 patients, the prevalence of ILD in patients with pSS was 13% [18]. Another meta-analysis by Joy and colleagues in 2023 aimed to investigate the prevalence, imaging patterns, and risk factors of ILD in connective tissue disease, and the prevalence of ILD in patients with pSS was 17% [63]. In 2023, in a meta-analysis conducted by Berardicurti and colleagues to investigate the prevalence of ILD in pSS, the combined prevalence of pSS-ILD was 23% [60]. In the current meta-analysis, which is the most up-to-date meta-analysis conducted in this field, the prevalence of ILD in patients with pSS has increased compared to previous meta-analyses, and considering the impact that ILD has on the quality of life and mortality of patients, this issue is very concerning.

Strengths of the current study include: )1) In the previous meta-analysis (Berardicurti), the databases PubMed, Embase, and Cochrane were searched until December 2022. While in the current meta-analysis, the databases ProQuest, PubMed, Web of Science, Cochrane, and Embase were searched until July 2024. )2) The number of studies and sample size in the current meta-analysis were greater than the number of studies and sample size in the previous meta-analysis (Berardicurti). )3) the prevalence of ILD in patients with pSS was presented by age and gender of patients, which was not present in the previous meta-analysis (Berardicurti). )4) the prevalence of NSIP and UIP, which were presented in the previous meta-analysis, the prevalence of LIP and OP was also reported in patients with pSS. )5) the results of the prevalence estimate of ILD in patients with pSS were reported by type of studies to reduce heterogeneity.

## Conclusions

One in four patients with pSS has ILD. Since ILD has degrees and is in mild and asymptomatic stages, its identification is not easy, even this prevalence may be higher. On the other hand, the prevalence of ILD was higher in men than in women. For this reason, men with pSS are more at risk of ILD than women. Also, the prevalence of ILD in the age group of 50 to 59 years was higher than other groups. In addition, the most common type of ILD is NSIP, and almost one in two people is affected by NSIP. As you can see, the statistics are very high and worrying. Therefore, it is recommended that patients with pSS, especially men and people who are 50 to 59 years old, be screened for ILD, as they are considered a high-risk group.

## Conflict of Interest

The authors declare that they have no conflict of interest.
